# Development and evaluation of height diameter at breast models for native Chinese Metasequoia

**DOI:** 10.1371/journal.pone.0182170

**Published:** 2017-08-17

**Authors:** Mu Liu, Zhongke Feng, Zhixiang Zhang, Chenghui Ma, Mingming Wang, Bo-ling Lian, Renjie Sun, Li Zhang

**Affiliations:** 1 Precision Forestry Key Laboratory of Beijing, Beijing Forestry University, Beijing,China; 2 The Key Laboratory for Silviculture and Conservation of Ministry of Education, Beijing Forestry University, Beijing,China; 3 School of Nature Conservation, Beijing Forestry University, Beijing China; 4 Northeast Forestry University, Harbin, China; Pacific Northwest National Laboratory, UNITED STATES

## Abstract

Accurate tree height and diameter at breast height (dbh) are important input variables for growth and yield models. A total of 5503 Chinese Metasequoia trees were used in this study. We studied 53 fitted models, of which 7 were linear models and 46 were non-linear models. These models were divided into two groups of single models and multivariate models according to the number of independent variables. The results show that the allometry equation of tree height which has diameter at breast height as independent variable can better reflect the change of tree height; in addition the prediction accuracy of the multivariate composite models is higher than that of the single variable models. Although tree age is not the most important variable in the study of the relationship between tree height and dbh, the consideration of tree age when choosing models and parameters in model selection can make the prediction of tree height more accurate. The amount of data is also an important parameter what can improve the reliability of models. Other variables such as tree height, main dbh and altitude, etc can also affect models. In this study, the method of developing the recommended models for predicting the tree height of native Metasequoias aged 50–485 years is statistically reliable and can be used for reference in predicting the growth and production of mature native Metasequoia.

## Introduction

Tree height and dbh are the two most important factors in surveys, production and management of forest resources and research on forest ecosystems [1,2]. They are usually used to calculate the volume, site index, forest growth and yield [3]; and to estimate forest volume, biomass and carbon stock [4]. Accurate tree height and dbh are necessary conditions for evaluating biomass and are of great importance for the research of forest growth models based on physiological ecology.

Compared with dbh, the observation of tree height is often affected by the complexity of the distribution of forest vegetation, forest density and landform [5,6]. At the same time, it takes time and effort to measure tree height since there are some limitations caused by observational error and visual disturbance [7], which increases the cost of the forest survey. Therefore, it is very necessary to construct a simple and accurate tree height-dbh model to estimate the height of trees [8].

The allometry equation of tree height and dbh is usually used in estimating tree height. This method neglects the possible large deviation in estimating biomass by allometry [9]. There are currently many studies on tree height-dbh models, and some tree height-dbh models of common tree species have achieved good effects in application [10–12]. These studies mainly focus on artificial forests, natural forests and pure forests [13–17]. An individual tree growth model is the basis of a forest growth and production forecast [18]. Huang et al. [11] evaluated the main species of Alberta by using 20 non-linear tree height-dbh models. The non-linear function fitting is relatively easier, so the non-linear tree height-dbh functions are widely used in the prediction of tree height [19–22]. However, changes in tree height and dbh may lead to a deviation in the predicted tree height. In tree height and dbh relationships, there is a correlation between tree height and growing conditions, forest density, tree age, basal area and dominant tree height and dbh. The correct choice of allometry models is the key in accurate prediction [23]. In this process, the choice of parameters is a key factor in making errors in models. The proper parameters can improve the data fitting of models [24]. Therefore, we cannot apply the same function to all the tree height-dbh models.

Metasequoia is a relict plant and is known as a “living fossil”. The earliest Metasequoia fossil plants appeared in the sedimentary formation in the Mesozoic Cretaceous period, which was 63~110 million years ago [25]. The ancient Metasequoia plant originated in the Arctic Circle and then gradually expanded southward. In the quaternary Pleistocene period, when temperature dropped dramatically, Metasequoia plants gradually disappeared [26].The native population of existing Metasequoia is distributed only at the junction of the Hubei, Hunan, Sichuan and Chongqing Provinces of China. Metasequoia in other regions were directly or indirectly introduced from the studied area. This area has become the only existing shelter for the native population of Metasequoia [27].

The tree population of native Metasequoia is one of the 69 endangered species in China [28]. It is the only existing habitat of the most primitive population of Metasequoia, therefore is the only place to study the living situation of the natural population of Metasequoia which preserves the most complete gene pool of the existing Metasequoia and is of great scientific value. This plays an irreplaceable role in the study of the genetic diversity and other biological characteristics of Metasequoia. The study of the native population and its living environment is of great significance to paleobotany, paleoclimatology and paleogeology [29].

Up to now, there has not been any study worldwide on the allometry models of tree height and dbh of native Chinese Metasequoia. This thesis aims to study the allometry equation of native Metasequoia by using tree height and dbh data. It is significant in clarifying the development and structure of this species. Previous studies were mostly conducted on plantations, and the growth time was short. However, predicting the height of native Metasequoia by using area-based tree height-dbh models has high uncertainty. Therefore, this study aims to 1) develop a model that can be used to predict the height-dbh relationship of native Metasequoia in China and 2) determine which variables have a significant effect on the relationship between tree height and dbh.

## Materials and methods

### Ethics statement

Our study sites (Xingdoushan National Nature Reserve, Hubei) are owned by the National Forestry Bureau of Enshi Tujia and Miao Autonomous Prefecture. Our research work was conducted in collaboration with the National Forestry Bureau of Enshi Tujia and Miao Autonomous Prefecture. We are permitted by the Forestry Bureau to collect the related plant sample data. Our research object is Metasequoia, the local protected species.

### The profile of the study sites

**Fig 1:**
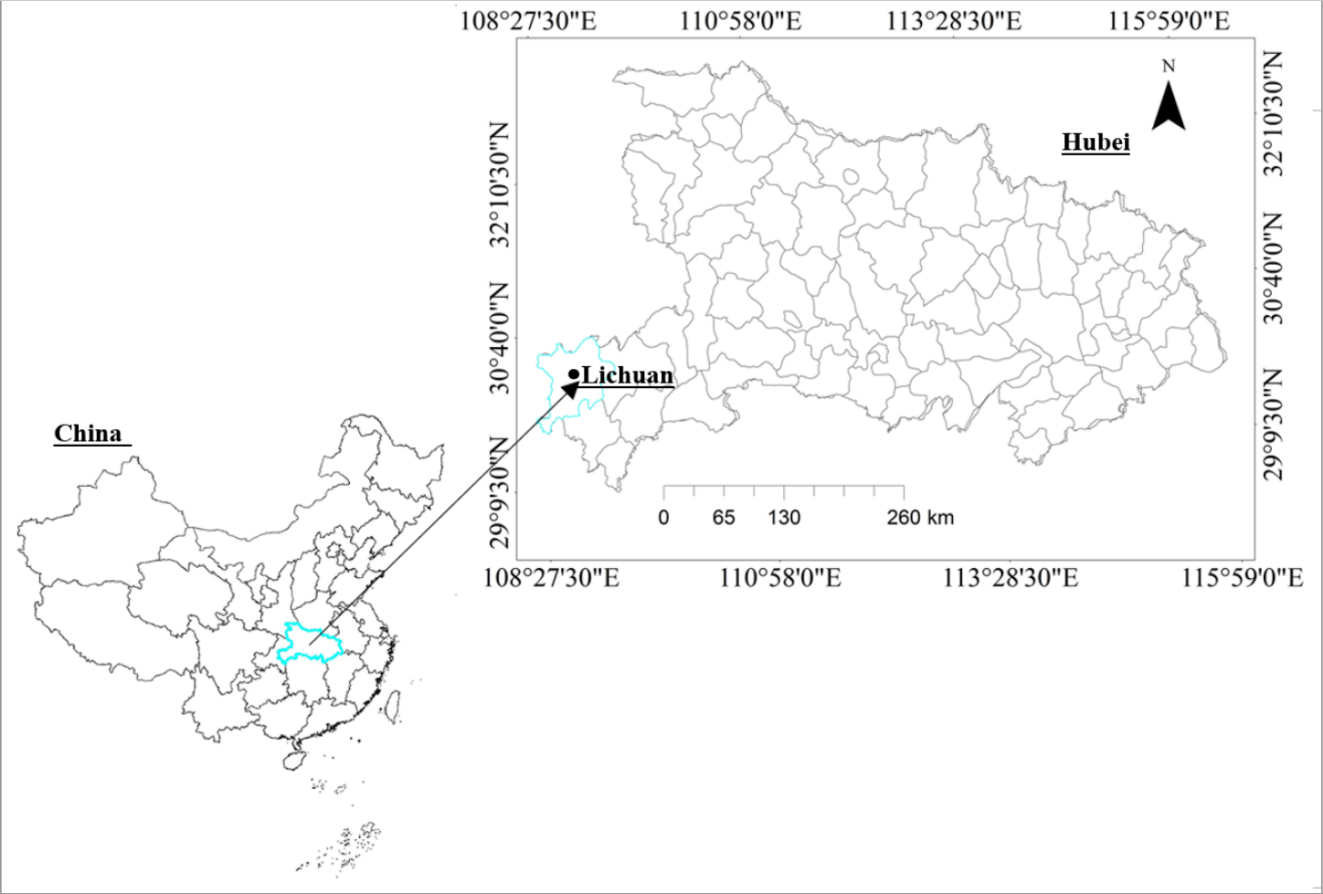
Geographic location of sampling sites. doi: 10.6084/m9.figshare.4956284.g001.

The study sites are located in Enshi City, Lichuan City and Xianfeng County of southwestern Hubei Province (Fig 1). The protection zone is divided into two districts: the eastern part is the Xingdou mountain area, located at the junction of Enshi City, Lichuan City and Xianfeng County, which is located at north latitude 29° 57' ~ 30° 10' and east longitude 108° 57' ~ 109° 27'; and the western part is the Xiaohe area, located in Lichuan City, which is located at north latitude 30° 04' ~ 30° 14' and east longitude 108° 31' ~ 108° 48', with a total area of 68339 hm^2^. The Metasequoia under study is in the Xiaohe area, the western part of the protection zone. The study sites are located in a sub-tropical continental monsoon area. The annual precipitation is 1481 mm. The annual average temperature is 14.9°C. The annual average frost-free period is 217 days. The trees of Metasequoia under study are distributed at an altitude of 900–1350 m. The soil in the study sites is loam, mainly a mixture of yellow soil and brown soil, with a small amount of purple soil. There are 5763 trees of native Metasequoia in total, and we collected data from all these trees. From the material collection and investigation, we know that the age of the Metasequoia trees is 50–510 years and that these trees are distributed in 4 towns, 16 administrative zones and 45 villages, with a total area of more than 800 km^2^. The trees are scattered, with a maximum of 20 in one district. The oldest trees are 510 years old, with a dbh of 248 cm and a height of 51 m. The average age of the trees is 97 years. The average dbh is 65.24 cm, and the average height is 27.84 m.

### Data collection

The data that were collected from the trees are individual measurements, including dbh, tree height, elevation and tree age, collected using a dbh ruler, height finder, Runtastic Altimeter PRO and vegetative cone, respectively. We chose the 10 toppest trees as the main samples. We also measured other stand-level variables, such as the dominant dbh (D_0_ cm), the dominant height (H_0_) and the basal area (BA). There are data from a total of 5746 Metasequoia trees available for this study. Some samples are either top dried, top broken, or struck by lightning, burned, or damaged by people. These samples could produce errors in the models; therefore, they were removed from the dataset. There were 5503 samples remaining that were studied.

The data from the 5503 sample trees were divided into two parts at random. The majority of the data (80%) was used for model calibration, and the remainder (20%) was used for verifying the consistency of the calibration data and the models. The calibration data show that the average dbh and height are 57.03 cm and 27.61 m, respectively. However, according to the validation data, the average dbh and height are 57.3 cm and 27.73 m, respectively. Table 1 shows the description of the stand-level variables.

**Table 1:** Regional Metasequoia sample statistics.

		h(m)	d(cm)	BA(cm^2^)	ASL(m)	T(y)	H_0_(m)	D_0_(cm)
Fitting data	Mean	27.61	57.03	2675.36	1187.16	95	43.04	122.59
N = 4401	Max	46.41	134.68	14246.35	1590	485	46.41	134.68
	Min	16.69	26.35	545.21	750	50	40.5	110.14
	Standard deviation	3.78	12.43	1221.36	111.38	32.58	3.26	8.79
Validation data	Mean	27.73	57.3	2686.72	1185.33	95.92	38.11	95.68
N = 1102	Max	40.09	109.8	9468.78	1605	325	40.09	109.8
	Min	18.8	28.47	636.69	856	50	37.12	95.68
	Standard deviation	3.55	11.75	1124.55	11.93	32.91	0.89	6.39

h: height; d: diameter at breast height (dbh); BA: basal area; ASL: Above Sea Level; T: age of the stand; H_0_: dominant height of the stand, m; D_0_: dominant dbh of the stand, cm; N: number of trees. doi: 10.6084/m9.figshare.4956284.t001

### Model selection and evaluation

We chose the linear and non-linear models and compared their performance. In this study, we selected two groups of general tree height-dbh models. There are 53 equations in total, as shown in Table 2. The criteria set for the two groups of models is in accordance with the number of variables, as follows:

Group 1: models that use one independent variable. We only need to measure the dbh.
Group 2: models that use two or more independent variables. We need to measure the dbh and other variables.

**Table 2:** Selected tree height-dbh models.

No.	Models	References	Group
Liner models			
1	*h* = *a*_0_ + *a*_1_*d*	[1]	1
2	*h* = *a*_0_ + *a*_1_log*d*	[10]	1
3	*h* = *a*_0_ + *a*_1_*d* + *a*_2_*d*^2^	[10]	1
4	*h* = *a*_0_ + *a*_1_*d*^2^ + *a*_2_*d*^3^	[10]	1
5	*h* = *a*_0_ + *a*_1_*d*^−1^ + *a*_2_*d*^2^	[10]	1
6	*h* = *a*_0_ + *a*_1_(*d*/*Dq*) + *a*_2_*Hm*	[1]	2
7	*h* = 1.3 + *a*_0_ + *a*_1_*log*(*d*/*Dq*) + *a*_2_*log*(*Hm*)	[1]	2
Non-linear models			
8	$h=1.3+a_{0}{\left(1-e^{-a_{1}d}\right)}^{a_{2}}$	[30]	1
9	$h=1.3+a_{0}d^{a_{1}}$	[31]	1
10	$h=1.3+e^{a_{0}+a_{1}/(d+1)}$	[29]	1
11	*h* = 1.3 + *a*_0_*d*/(*a*_1_ + *d*)	[21]	1
12	$h=1.3+a_{0}(1-e^{-a_{1}d})$	[32]	1
13	$h=1.3+10^{a_{0}}d^{a_{1}}$	[33]	1
14	*h* = 1.3 + *a*_0_*d*/(*d* + 1) + *a*_1_*d*	[34]	1
15	$h=1.3+a_{0}{\left(d/\left(1+d\right)\right)}^{a_{1}}$	[10]	1
16	$h=1.3+a_{0}/(1+a_{1}e^{-a_{2}d})$	[35]	1
17	$h=1.3+a_{0}{\left(1-e^{-a_{1}d}\right)}^{a_{2}}$	[36]	1
18	$h=1.3+a_{0}d^{a_{1}d^{-a_{2}}}$	[37]	1
19	$h=1.3+a_{0}e^{-a_{1}e^{-a_{2}d}}$	[38]	1
20	*h* = 1.3 + *d*^2^/(*a*_0_ + *a*_1_*d* + *a*_2_*d*^2^)	[10]	1
21	$h=1.3+a_{0}d^{a_{1}d^{a_{2}}}$	[39]	1
22	$h=1.3+a_{0}e^{a_{1}/d+a_{2}}$	[40]	1
23	$h=1.3+a_{0}/(1+{a_{1}}^{-1}d^{-a_{2}})$	[41]	1
24	*h* = 1.3 + *a*_0_ + *a*_1_/(*d* + *a*_2_)	[42]	1
25	$h=1.3+a_{0}e^{\left(-a_{1}d^{a_{2}}\right)}$	[43]	1
26	$h=1.3+a_{0}(e^{-e(-a_{1}(d-a_{2}))})$	[44]	1
27	$h=1.3+e^{\left(a_{0}+a_{1}d^{a_{2}}\right)}$	[20]	1
28	$h=a_{0}+e^{\left(a_{1}+a_{2}/d\right)}$	[45]	1
29	$h=1.3+a_{0}/(1+a_{1}d^{a_{2}})$	[46]	1
30	$h=1.3+a_{0}de^{-a_{1}d}$	[46]	1
31	$h=1.3+a_{0}d^{a_{1}e^{-a_{2}d}}$	[46]	1
32	$h=1.3+a_{0}e^{a_{1}/d+a_{2}}$	[46]	1
33	$h=1.3+{\left(a_{0}+a_{1}/d\right)}^{a_{2}}$	[46]	1
34	*h* = 1.3 + *d*^2^/(*a*_0_ + *a*_1_*d*)^2^	[22]	1
35	$h=1.3+a_{0}e^{\left(a_{1}/d\right)}$	[47]	1
36	$h=1.3+e^{\left(a_{0}+a_{1}/(d+1)\right)}$	[19]	1
37	$h=a_{0}+e^{\left(a_{1}+a_{2}/d\right)}$	[48]	1
38	$h=e^{\left(a_{0}+a_{1}d^{-0.5}+a_{2}d^{-1}+a_{3}d^{-2}\right)}$	[48]	1
39	$h=a_{0}+e^{\left(a_{1}+a_{2}d+a_{3}{\left(lnd\right)}^{2}\right)}$	[48]	1
40	$h=1.3+a_{0}{\left(BA\right)}^{a_{1}}(1-e^{-a_{2}d})$	[49]	2
41	$h=1.3+(H_{m}-1.3)e^{a_{0}(1-d/Dq)+(d/Dq-1/d)}$	[15]	2
42	$h=H_{0}(1+a_{0}e^{a_{1}H_{0}})(1-e^{-a_{2}d/H_{0}})$	[30]	2
43	$h=1.3+a_{0}{H_{0}}^{a_{1}}d^{a_{2}{H_{0}}^{a_{3}}}$	[50]	2
44	$h=1.3+(a_{0}+a_{1}H_{0}-a_{2}Dq)e^{-a_{3}/d}$	[51]	2
45	$h=1.3+(a_{0}+a_{1}H_{0}-a_{2}Dq)e^{-a_{3}/\sqrt{d}}$	[15]	2
46	$h=1.3+(a_{0}+a_{1}H_{0}-a_{2}Dq+a_{3}BA)e^{-a_{4}/\sqrt{d}}$	[15]	2
47	*h* = *a*_0_ + *a*_1_*Hm* + *a*_2_*Dq*^0.95^ + *a*_3_*e*^−0.08*d*^ + *a*_4_*Hm*^3^*e*^−0.08*d*^ + *a*_5_*Dq*^3^*e*^−0.08*d*^	[15]	2
48	$h=10^{\left(a_{0}+a_{1}/d+a_{2}/t+a_{3}/Dq^{t}\right)}$	[15], [52]	2
49	$h=e^{\left(a_{0}+a_{1}lnDq+a_{2}lnN+a_{3}\sqrt{d}\right)}$	[53]	2
50	$h=a_{0}+a_{1}Hm+a_{2}Dq+a_{3}e^{a_{4}d}+a_{5}Hm^{a_{6}}e^{a_{4}d}+a_{7}Dq^{a_{8}}e^{a_{4}d}$	[52]	2
51	$h=e^{\left(a_{0}+a_{1}lnH_{0}+a_{2}/t+a_{3}lnN/d+a_{4}/dt+a_{5}/d\right)}$	[15], [52]	2
52	$h=a_{0}{H_{0}}^{a_{1}}{\left(BA\right)}^{a_{2}}N^{a_{3}}e^{\left(a_{4}/t+a_{5}/d\right)}$	[54]	2
53	$h=1.3+(a_{0}+a_{1}Hm+a_{2}H_{0}){\left(1-e^{-a_{3}d}\right)}^{a_{4}}$	[55]	2

h: height, m; d: diameter at breast height (dbh), cm; BA: basal area, cm^2^; t: age of the stand; H_0_: dominant height of the stand, m;D_0_: dominant dbh of the stand, cm; H_m_: mean height; Dq: quadratic mean dbh; a_0_-a_8_ are parameters. The base is 10 for logarithm; N is number of trees. doi: 10.6084/m9.figshare.4956284.t002.

Model selection and evaluation are based on the graphical and numerical data analysis, as follows: (1) root of mean square error (RMSE) (Eq 1), used for the accuracy estimation of the analysis (the smaller, the better); (2) adjusted coefficient of determination ($R_{adj}^{2}$) (Eps.2 and 3), which reflects the part of the total variance explained by the model and takes into account the parameters that are necessary in making the estimation (the greater the value, the more interrelated between the variables); (3) deviation (Eq 4) and relative deviation (Eq 5), which is the deviation in the observed results from the evaluation model (the smaller, the better the effect); and (4) Akaike’s information criterion (AIC) (Eqs 6 and 7), which is an information standard commonly used to choose the best model (as a rule, the model whose AIC value is lower is preferred)[5]. The expressions of the statistics are shown as follows:

$$Root\;of\;mean\;square\;error\;RMSE=\sqrt{\frac{\overset{n}{\underset{i=1}{\sum }}{\left(h_{i}-{\hat{h}}_{i}\right)}^{2}}{n-p-1}}$$ (1)

$$\mathrm{Coefficient}\;\mathrm{of}\;\mathrm{determination}:\;R^{2}=1-\frac{\overset{n}{\underset{i=1}{\sum }}{\left(h_{i}-{\hat{h}}_{i}\right)}^{2}}{\overset{n}{\underset{i=1}{\sum }}{\left(h_{i}-{\bar{h}}_{i}\right)}^{2}}$$ (2)

$$Adjusted\;coefficient\;of\;determination:\;R_{adj}^{2}=1-(1-R^{2})*\frac{n-1}{n-p-1}$$ (3)

$$Bias=\frac{\overset{n}{\underset{i=1}{\sum }}\left({\hat{h}}_{i}-h_{i}\right)}{n}$$ (4)

$$Relative\;bias(\%)=\frac{\overset{n}{\underset{i=1}{\sum }}({\hat{h}}_{i}-h_{i})/h_{i}}{n}*100$$ (5)

$$Residual\;sum\;of\;squares:\;RSS=\sum_{i=1}^{n}{\left(h_{i}-{\hat{h}}_{i}\right)}^{2}$$ (6)

Akaike’sinformationcriterionAIC=nln⁡(RSS)+2(p+1)−nln⁡(n) (7)

Where *h*_*i*_ is the observation value; ${\hat{h}}_{i}$ is the forecast value and; ${\bar{h}}_{i}$ is the average value; *n* is the total number of data used to the fitted model; and *p* is the number of independent variables.

### Data analysis

Because many of the selected models were non-linear, they were fitted with the statistical package SPSS 20.0 (SPSS for Windows version 20.0) and the Levenberg-Marquardt (LM) methods. The initial values of the parameters for starting the iterative procedure were used to fit the data. The regression analysis was made using the SPSS program.

## Results

### The relationship between tree height and dbh

**Fig 2:**
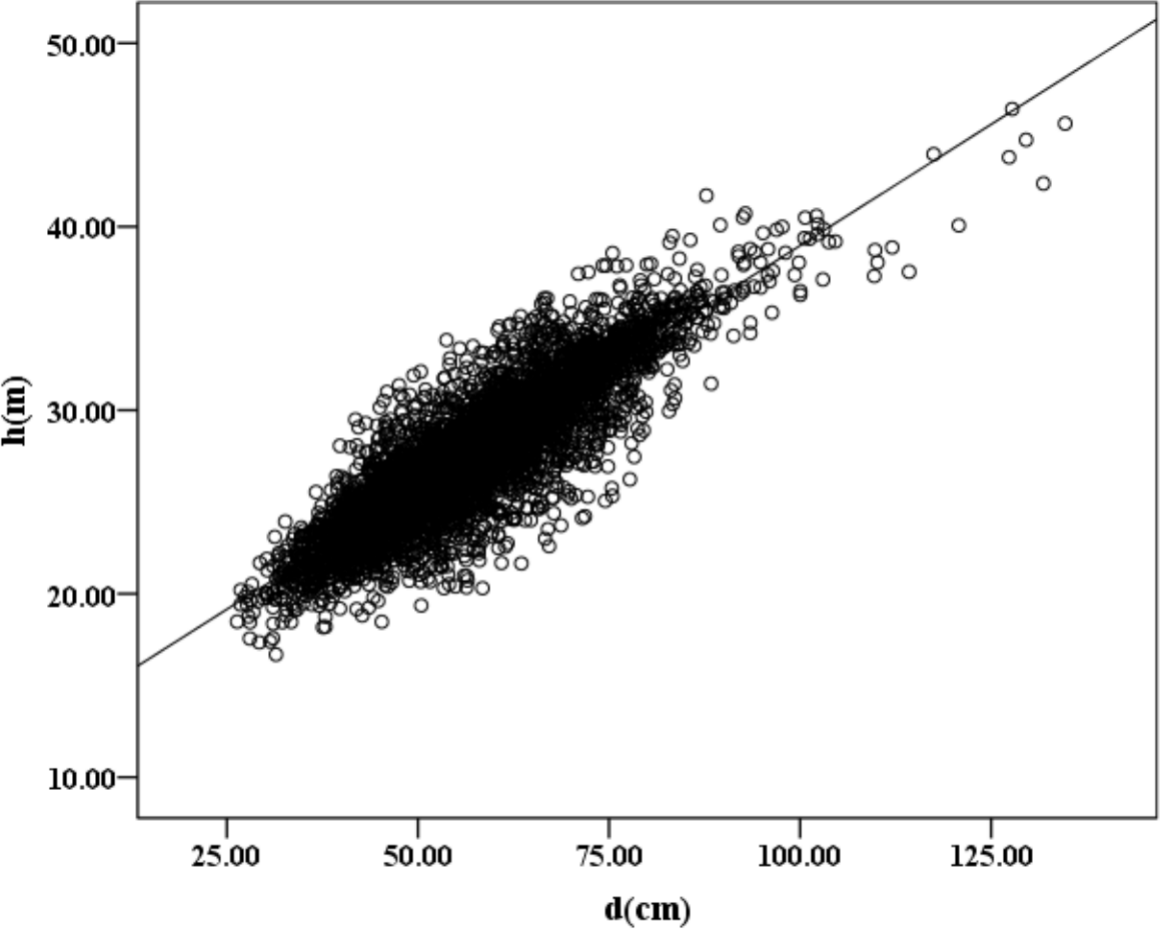
Scatter diagram of the tree height and dbh of a single Metasequoia tree. doi: 10.6084/m9.figshare.4956284.g002.

As shown in Fig 2, which is a scatter diagram of the tree height and dbh of all the 5503 native Metasequoia, the minimum dbh is greater than 26 cm, and with the increase in the dbh, the tree height also slowly increases. The solid line is the trend line, whose formula is *h* = 12.546 + 0.264 * *d*(*R*^2^ = 0.7582).

### Model fitting and selection

Tables 3 and 4 are the prediction and fitting results of the fitting data and validation data from Group 1 models and Group 2 models, respectively. The two groups of models show good results, except for models 44, 47 and 48. Therefore, we can see that these three models are not fit for the prediction of the data, and we will remove their results in later statistics. As shown in Table 3, in the Group 1 models, the changes of the derived value between the adjusted *R*^2^ and RMES are roughly equivalent. In the Group 1 models, model 4 is the most suitable linear model (the adjusted *R*^2^ value is the highest, and the RMES value, deviation sum and AIC are the lowest). It derives the most accurate result in the fitting of calibration data and is suitable for estimating the height of most trees in Group 1. Model 22 is the most suitable non-linear model. Both models have only one independent variable (dbh) that is used to predict tree height, and their derived data are in good agreement. When considering the other variables (Group 2 models) the models show different performances. In the calibration data from the Group 2 models, the derived *R*^2^ value is 0.7456~0.7737, and the RMSE value is 1.7965~1.9050 (Table 4). In addition, most of the Group 2 models have greater adjusted *R*^2^ values and smaller RMSE values than the Group 1 models. In the models of Group 1, the calibration dataset of the AIC value is relatively higher. The calibration data of the AIC value in Group 2 models is smaller than that in the Group 1 models. The choice of models should be based on the arrangement of the expression of models using calibration data and validation data (the adjusted *R*^2^, RMSE, the absolute deviation, the relative deviation and the AIC). In this analysis, the top two or three models are summarized below in Table 5 (the numbers in brackets indicate the ranking of the parameters). The model with the adjusted *R*^2^ value closest to the highest value and a deviation (absolute and relative deviation) closest to zero is thought to be the best. The lower the value of RMSE and AIC, the higher the ranking of the model. For each model, its final ranking is the sum of the statistics of the five evaluation values. The model with the minimum sum (i.e., the highest ranking) is considered to be the functional model that is most suitable for estimating the growth of Metasequoia. According to this analysis, in the models of Group 1, the calibration data show that model 22 is the most suitable, and the validation data show that model 22 and model 4 are both the most suitable. In the Group 2 models, model 52 ranks the same according to both calibration and validation data. Overall, model 4 is the most suitable of the linear models and model 52 is the most suitable of the non-linear models. In the Group 1 models, model 22 is the most suitable. In all models, model 52 and model 51 are the most suitable non-linear models; model 4 is the most suitable linear model. Overall, the non-linear models performed slightly better than the linear models.

**Table 3:** Calibration data and validation data adaptive statistics for models in Group 1. doi: 10.6084/m9.figshare.4956284.t003.

No.	Variables	Fitting data				Validation data			
		Bias(m)	RMSE(m)	AIC	Adjusted *R*^2^	Bias(m)	RMSE(m)	AIC	Adjusted *R*^2^
Liner models									
1	h,d	0.0000	1.8411	5375.1	0.7623	0.0000	1.8087	1311.3	0.7397
2	h,d	0.0000	1.8902	5606.7	0.7495	0.0000	1.8578	1370.4	0.7253
3	h,d	0.0000	1.8371	5356.2	0.7634	0.0000	1.8086	1311.2	0.7397
4	h,d	0.0000	1.8329	5353.6	0.7669	0.0000	1.8074	1309.8	0.7400
5	h,d	0.0000	1.8497	5416.0	0.7601	0.0000	1.8135	1317.2	0.7383
Non-linear models									
8	h,d	-0.0015	1.8419	5378.9	0.7621	-0.0017	1.8176	1322.1	0.7371
9	h,d	-0.0015	1.8419	5378.9	0.7621	-0.0017	1.8176	1322.1	0.7371
10	h,d	-0.0075	1.9460	5862.8	0.7345	-0.0057	1.9021	1422.3	0.7121
11	h,d	-0.0085	1.8717	5520.4	0.7544	-0.0068	1.8445	1354.5	0.7292
12	h,d	-0.0155	1.8906	5608.5	0.7494	-0.0119	1.8596	1372.6	0.7248
13	h,d	-0.0015	1.8419	5378.9	0.7621	-0.0017	1.8176	1322.1	0.7371
14	h,d	0.0001	1.8403	5371.5	0.7625	0.0000	1.8087	1311.3	0.7397
15	h,d	-0.0076	1.9490	5876.2	0.7337	-0.0058	1.9044	1425.1	0.7114
16	h,d	0.0001	1.8369	5355.3	0.7634	-0.0001	1.8076	1310.0	0.7400
17	h,d	-0.0015	1.8419	5378.9	0.7621	-0.0017	1.8176	1322.1	0.7371
18	h,d	0.0000	1.8441	5389.3	0.7616	-0.0022	1.8090	1311.6	0.7396
19	h,d	-0.0004	1.8368	5354.5	0.7635	-0.0001	1.8081	1310.5	0.7398
20	h,d	0.0000	1.8380	5360.4	0.7631	-0.0003	1.8090	1311.7	0.7396
21	h,d	0.0000	1.8378	5359.6	0.7632	0.0000	1.8090	1311.6	0.7396
22	h,d	0.0000	1.8280	5288.5	0.7693	0.0000	1.8074	1309.7	0.7400
23	h,d	-0.0001	1.8419	5379.0	0.7621	-0.0017	1.8095	1312.3	0.7394
24	h,d	0.0000	1.8373	5356.9	0.7633	0.0000	1.8086	1311.2	0.7397
25	h,d	0.0000	1.8379	5359.7	0.7632	0.0000	1.8090	1311.7	0.7396
26	h,d	0.0000	1.8368	5354.5	0.7635	-0.0001	1.8081	1310.5	0.7398
27	h,d	0.0000	1.8379	5359.7	0.7632	0.0000	1.8090	1311.7	0.7396
28	h,d	0.0000	1.8405	5372.2	0.7625	0.0000	1.8094	1312.2	0.7395
29	h,d	-0.0015	1.8419	5378.9	0.7621	0.0000	1.8095	1312.3	0.7394
30	h,d	-0.0249	1.8419	5378.9	0.7621	-0.0182	1.8743	1389.9	0.7204
31	h,d	0.0000	1.8385	5362.9	0.7630	0.0000	1.8083	1310.8	0.7398
32	h,d	0.0000	1.8371	5355.9	0.7634	0.0000	1.8084	1310.9	0.7398
33	h,d	-0.0003	1.8387	5363.7	0.7630	-0.0002	1.8104	1313.4	0.7392
34	h,d	-0.0090	1.9069	5684.3	0.7450	-0.0069	1.8719	1387.1	0.7211
35	h,d	-0.0078	1.9520	5889.8	0.7329	-0.0059	1.9068	1427.9	0.7106
36	h,d	-0.0075	1.9460	5862.8	0.7345	-0.0057	1.9021	1422.3	0.7121
37	h,d	0.0000	1.8405	5372.2	0.7625	0.0000	1.8094	1312.2	0.7395
38	h,d	0.0000	1.8370	5355.4	0.7634	0.0000	1.8081	1310.6	0.7398
39	h,d	0.0000	1.8368	5354.5	0.7635	0.0000	1.8076	1309.9	0.7400

**Table 4:** Calibration data and validation data adaptive statistics for models in Group 2. doi: 10.6084/m9.figshare.4956284.t004.

No.	Variables	Fitting data				Validation data			
		Bias(m)	RMSE(m)	AIC	*R*^2^	Bias(m)	RMSE(m)	AIC	*R*^2^
Liner models									
6	h,d,Dq,Hm	0.000	1.8411	5379.1	0.7623	0.0000	1.8087	1315.3	0.7397
7	h,d,Dq,Hm	0.000	1.8902	5610.7	0.7495	0.0000	1.8578	1374.4	0.7253
Non-linear models									
40	h,d,BA	-0.0015	1.8419	5380.8	0.7621	-0.0017	1.8176	1324.1	0.7371
41	h,d,Dq,Hm	0.0079	1.9050	5677.3	0.7456	0.0028	1.8286	1337.4	0.7339
42	h,d,H_0_	-0.0159	1.9013	5660.4	0.7465	-0.0121	1.8680	1384.5	0.7223
43	h,d,H_0_	-0.0015	1.8419	5380.9	0.7621	-0.0017	1.8176	1324.1	0.7371
44	h,d,H_0_,Dq	-0.0078	1.9520	5891.8	0.7329	-0.0059	1.9068	1429.9	0.7106
45	h,d,H_0_,Dq	-0.0048	1.8807	5564.6	0.7520	-0.0037	1.8510	1364.3	0.7274
46	h,d,H_0_,Dq,BA	-0.0003	1.8426	5388.4	0.7619	-0.0001	1.8116	1320.8	0.7388
47	h,d,Dq,Hm	0.0000	2.5633	8291.3	0.5393	0.0000	2.4307	1967.4	0.5298
48	h,d,t	-0.4314	4.2983	12790.1	0.2850	-0.5747	4.7089	3426.1	0.2646
49	h,d,Dq,N	0.0019	1.8341	5321.5	0.7633	0.0000	1.8075	1309.8	0.7397
50	h,d,Dq,Hm	0.0000	1.8372	5360.7	0.7633	0.0000	1.8086	1315.2	0.7397
51	h,d,H_0_,N,t	0.0000	1.8337	5321.2	0.7636	0.0000	1.7609	1308.2	0.7532
52	h,d,BA,N,t	0.0000	1.7965	5165.4	0.7737	0.0000	1.7198	1206.1	0.7646
53	h,d,H_0_,Hm	-0.0015	1.8419	5382.9	0.7621	-0.0017	1.8176	1326.1	0.7371

**Table 5:** Model ranking based on performance. doi: 10.6084/m9.figshare.4956284.t005.

	Group 1 models		Group 2 models		All models	
	Fitting data	Validation data	Fitting data	Validation data	Fitting data	Validation data
RMSE	22(1);4(2);19, 26,39(3)	22,4(1);16,39(2);19,26,38(3)	52(1);51(2);49(3)	52(1);51(2);49(3)	52(1);22(2);51(3)	52(1);51(2);4,22(3)
AIC	22(1);4(2);19, 26,39(3)	22(1);4(2);39(3)	52(1);51(2);49(3)	52(1);51(2);49(3)	52(1);22(2);51(3)	52(1);51(2);4,22(3)
*R*^2^	22(1);4(2);19, 26,39(3)	4,22,39,16(1);19,26,38,31,32(2)	52(1);51(2);6,49,50(3)	52(1);51(2);49(3)	52(1);22(2);51(3)	52(1);51(2);4,22(3)
Absolute bias	1–5,14,16,18–28,31–33,37–39	1–5,14,21,22,24,25,27–29,31,32,37–39	47,50,51,52,6,7,49,41(1)	49,50,51,52,6,7,47(1)	1–7,14,16,18,20–22,24–28,31,32,37–39,41,47,49-52(1)	1–7,14,21,22,24,25,27–29,31,32,37–39,41,49,50-52(1)
Relative bias	30(1);12(2);11(3)	24(1);23(2);18(3)	42(1);51(2);52(3)	42(1);51(2);52(3)	30(1);12,42(2);51(3)	21(1);42,23(2)18,51(3)

**Fig 3:**
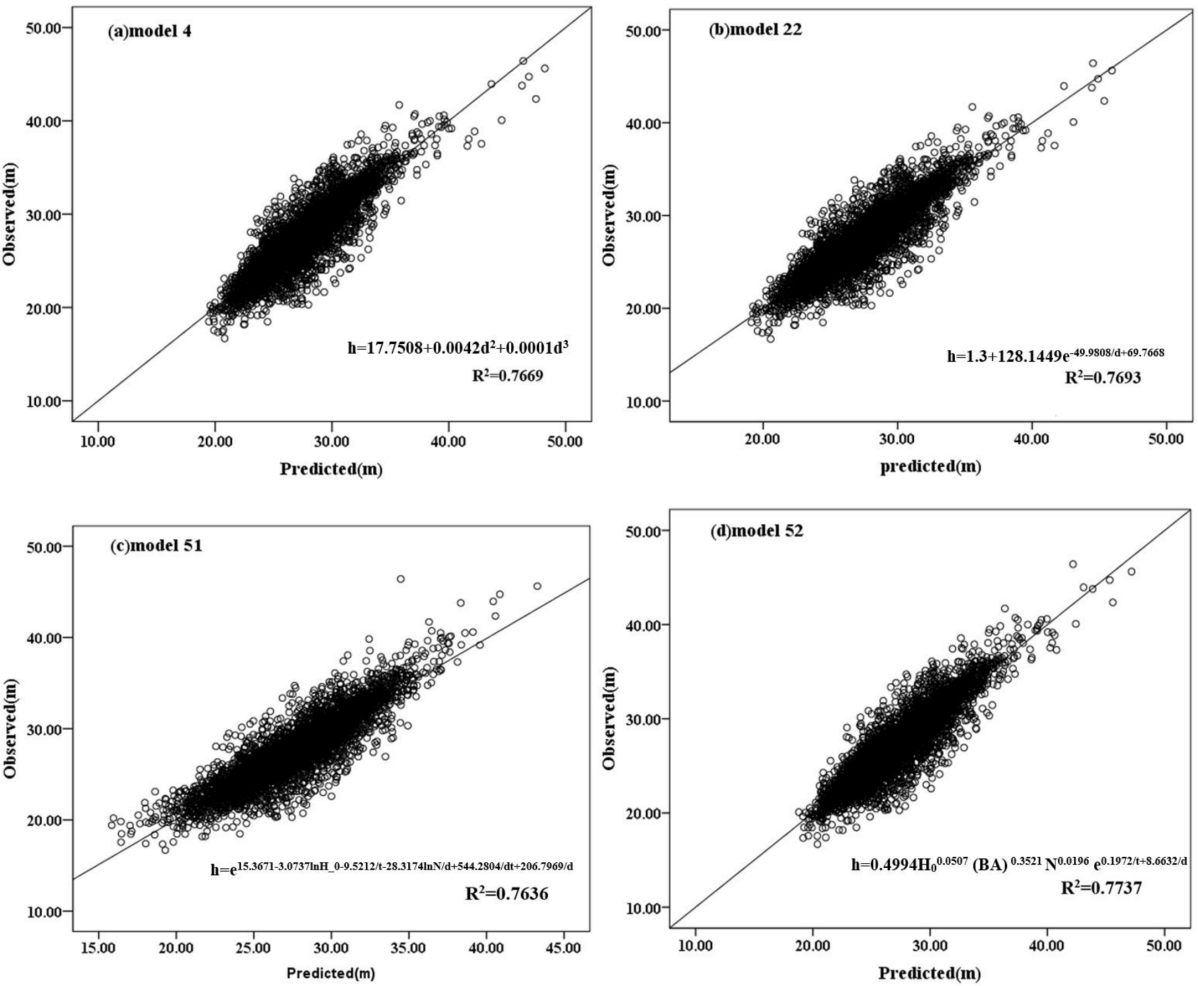
Graph of predicted values in contrast to observed values in the calibration dataset for the three best models (a: Model 4; b: Model 22; c: Model 51; d: Model 52). The solid line represents the diagonal. doi: 10.6084/m9.figshare.4956284.g003.

**Fig 4:**
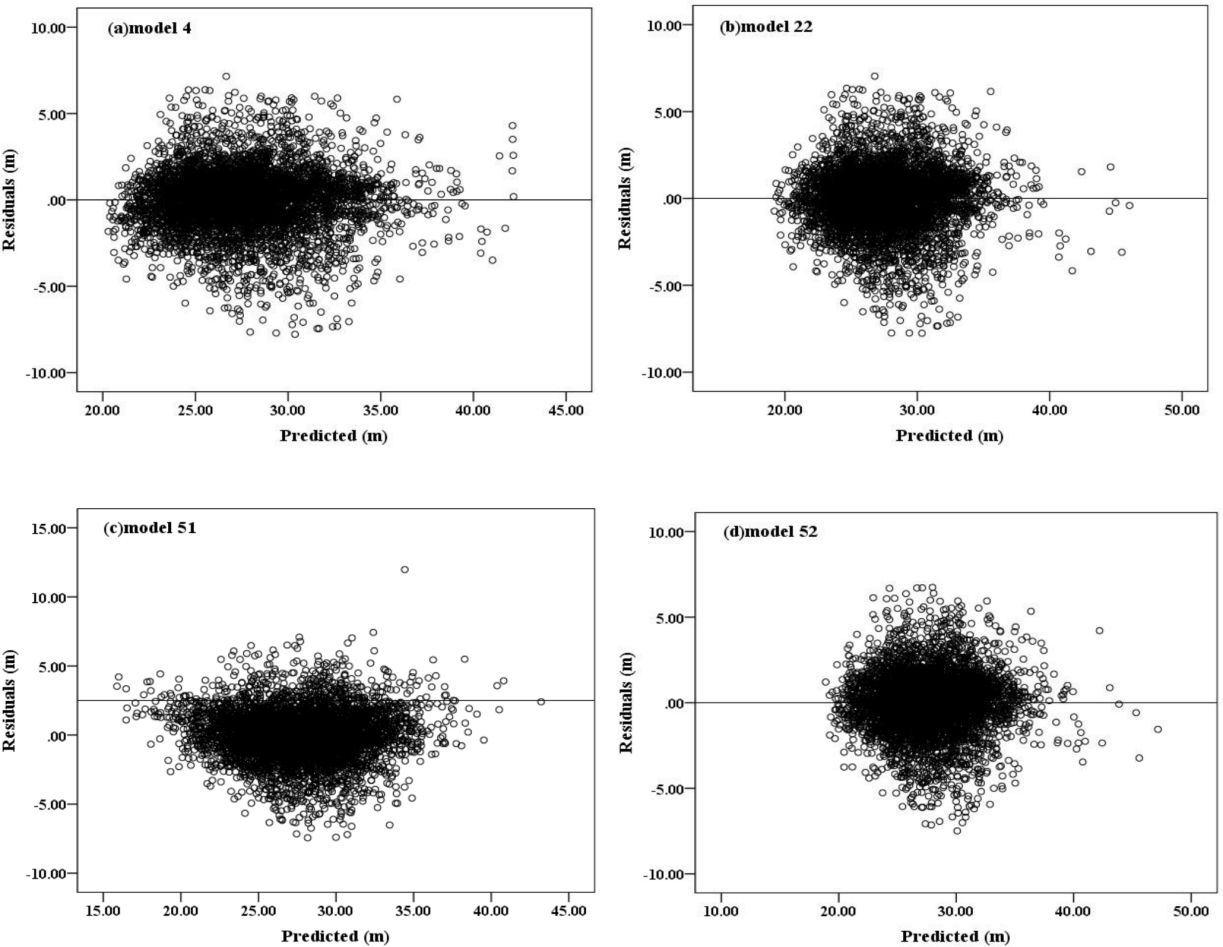
Residual plots in the calibration dataset for the three best models (a: Model 4; b: Model 22; c: Model 51; d: Model 52). doi: 10.6084/m9.figshare.4956284.g004.

**Fig 5:**
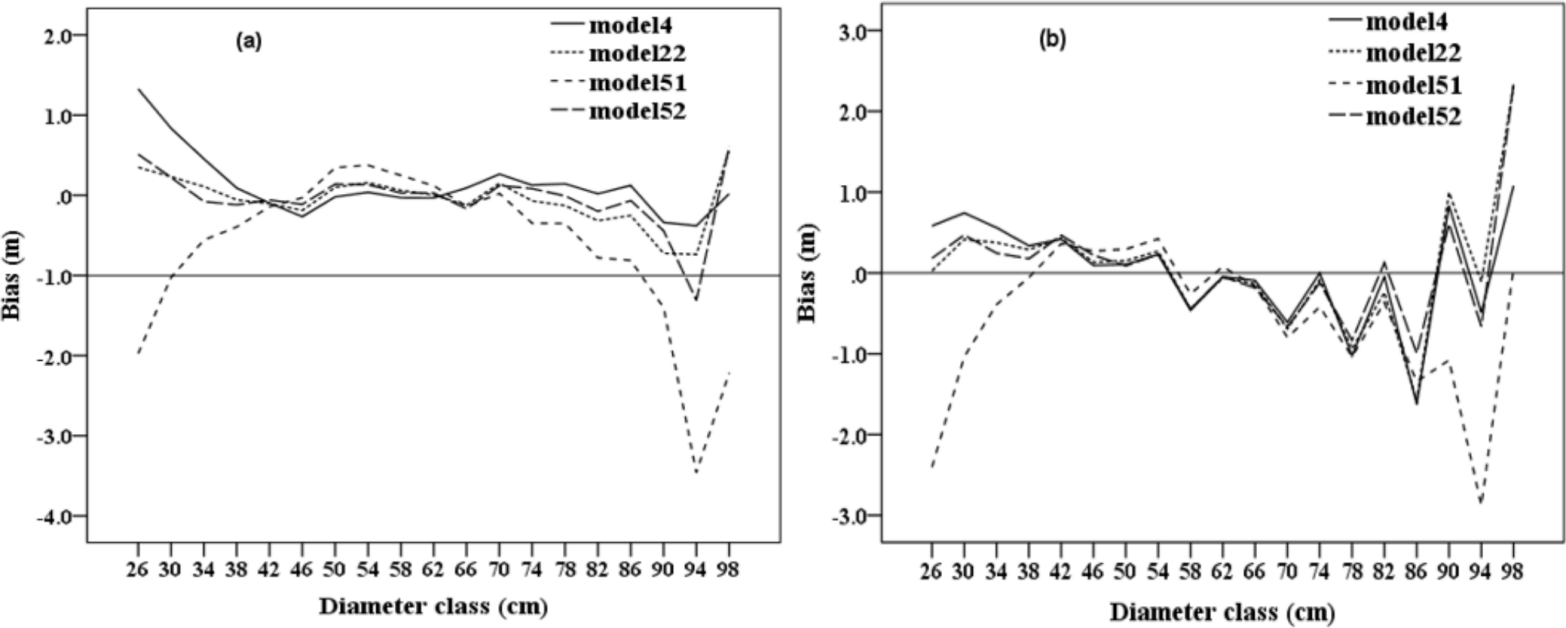
Values of average deviation in relation to dbh in the calibration and validation datasets for the two best models (a: calibration data; b: validation data). doi: 10.6084/m9.figshare.4956284.g005.

Fig 3 shows the predicted values and the observed values obtained by using the model. The straight line between the observed value and the predicted value is the determining factor of the model evaluation standard. Each model has a higher *R*^2^ value, so the solid line is closely surrounded by data points. There is no obvious tendency to overestimate or underestimate the observed height values. Fig 4 shows the residual error of the predicted height obtained from model 4, 22, 51 and 52 by using calibration data. Most data points are distributed around the solid line. These models all show the uniform variance of the predicted value, as well as the independence of the residuals. Fig 5 shows the average deviation value obtained from the diameter by models 4, 22, 51 and 52 using calibration data and validation data. From the above analysis, models 4, 22, 51 and 52 become the best four models in the study. However, the number of parameters in models 51 and 52 is higher than that in models 4 and 22, but with a higher *R*^2^ and a lower RMSE and AIC. Therefore, in the case that only the dbh is known, models 4 and 22 can predict tree height; and if tree age, dominant tree height, basal area and the number of samples are known, models 51 and 52 can be used to obtain a more accurate tree height.

### Parameter estimation

Initially, the model estimates all the parameters using the calibration data. Table 6 shows the parameter estimation and fitting statistics of models 4, 22, 51 and 52 using all data. All the parameters were significant (P < 0.05). It is easy to obtain the parameters of each model through calculations.

**Table 6:** Parameter estimaties and fitting statistics of the final models by using all data. doi: 10.6084/m9.figshare.4956284.t006.

Parameter	Model 4	Model 22	Model 51	Model 52
a_0_	17.7508	128.1449	15.3671	0.4994
a_1_	0.0042	-49.9808	-3.0737	0.0507
a_2_	0.0001	69.7668	-9.5212	0.3521
a_3_			-28.3174	0.0196
a_4_			544.2804	0.1972
a_5_			206.7969	8.6632
Adjusted *R*^2^	0.7583	0.7591	0.7314	0.7592
RMSE	1.8277	1.8237	1.8186	1.7806
Absolute bias (m)	0.0000	-0.0032	0.0001	0.0000
Relative bias (%)	0.4542	0.3904	0.4104	0.4630

## Discussion

### Evaluation and application of models

The choice of the models is based on the degree of optimization, the accuracy and the practical application. The models were compared and ranked after implementation (Table 5). Under this approach, models from Group 1 cannot be clearly differentiated. As shown in Fig 2, the result is that the tree height increases monotonically with the increase in the dbh. A reasonable tree height-dbh model should be consistent with the first order monotone increasing characteristic [56]. In the models of Group 2, the calibration and validation data for models 22, 51, and 52 are in agreement. Usually, the increase in the new independent variables can reduce the error in the height-dbh model and increase the accuracy of the model. Therefore, through large amounts of sampling and measuring the different variables, we derived the Group 2 models that are more suitable for the prediction of tree height than the Group 1 models.

In this research, model 4 is the only linear model with 2 variables of tree height and dbh, and the others are non-linear models, in which model 22 has 2 variables of tree height and dbh; model 51 has 5 variables of tree height, dbh, main tree height, sample numbers and tree age; and model 52 has 5 variables of tree height, dbh, basal area, sample numbers and tree age. The non-linear models are more accurate. Therefore, we believe that the non-linear models perform better than the linear models in the case of Metasequoia. The non-linear models are more flexible. The forms and parameters of the models are very important for the application of models. We have demonstrated the stability of the structure of the four models. Therefore, the parameter is the determining factor that affects the accuracy of the models in their application.

The development of simple and accurate models makes it possible for forestry workers to predict the height of a tree by relying on the diameter data of a region, which is very important for forest management. In this study, models 51 and 52 are similar. The four models are all consistent with statistical principles. There are a relatively larger number of parameters in models 51 and 52, and the increase in the number of parameters can improve the accuracy of the model in the prediction of tree height. The choice of the four models is not only statistically reliable but is easier for application. The practical application of the models is necessary for the prediction of tree height.

### The relationship between tree height and the study area variables

According to the study, bringing the study area variables as independent variables into the height-dbh model can improve the accuracy of tree height predictions [49]. The study area variables mainly include the development of comprehensive factors, such as the dominant tree height, dbh, tree age, basal area, stand density and growth status, etc. In the Group 1 models, only one independent variable of dbh (d) can be used to predict the relationship between height and dbh. Depending on the specific circumstances, if the other variables of the study area can be measured, Group 2 models are the best method. We can accurately assess the direct relationship between tree height and dbh using Group 2 models and can predict the future development.

A significant difference exists in the growth rate of tree height and dbh, and there is an allometric relationship between them. Because the average age of the Metasequoia trees collected in this study is more than 50 years and they are a mature forest, the tree height and dbh growth relationships are relatively stable. Although tree age is not the most important variable in the study of the tree height-dbh relationship, some differences in parameter estimation can be discovered when selecting models. Therefore, considering tree age when selecting models and parameters can make the tree height prediction more accurate.

## Conclusion

This study compared and predicted the native population of Metasequoia aged 50–485 in China using 53 height-dbh models. The choice of models is based on the principles of good fit, high accuracy and suitability for practical application. The results show that the composite models which include additional variables can improve the quality of the models. Linear model 4 and non-linear model 22, which have 3 parameters and 2 variables each, and non-linear models 51 and 52, which have 6 parameters and 5 variables each, can better predict the height of natural Metasequoia trees. Although stand age is not the most significant variable in the relationship between height and dbh, knowing the tree age can improve the accuracy of natural Metasequoia tree height prediction. In addition, sample size can also improve the accuracy of tree height prediction to some degree. In this study, forest density will not affect the growth of the trees. However, as a referential maximum value of growth of local Metasequoia, the main tree height can also improve the accuracy of model prediction. In remote or individual regions, because different ecological niches may have an important impact on the estimation of height, it is not suitable for application. In our analysis, the measurement error due to the ground level was not considered. This is because these errors likely cannot affect the allometric models [57].

This research aims to build some models to facilitate the prediction of tree height of local Metasequoia, thus making it easier to evaluate the growth of native Metasequoia and protect it as an important plant resource. Therefore, the sanctuary workers who have mastered the original data of local Metasequoia can predict tree height using models 51 or 52. General scientific researchers, in the case that they have measured the dbh, can use models 4 or 22 to predict tree height. The methods and the models recommended in this study are based on statistics, which are reliable for forest growth and survival estimates and planting management for Chinese native Metasequoia. After verification, these models can be applied in other regions and to other tree species.
